# Supplementary figures and images for: The MODY-associated KCNK16 L114P mutation increases islet glucagon secretion and limits insulin secretion resulting in transient neonatal diabetes and glucose dyshomeostasis in adults
Source: eLife. 2024 May 3;12:RP89967. doi: 10.7554/eLife.89967 (PMC11068355; doi:10.7554/eLife.89967)

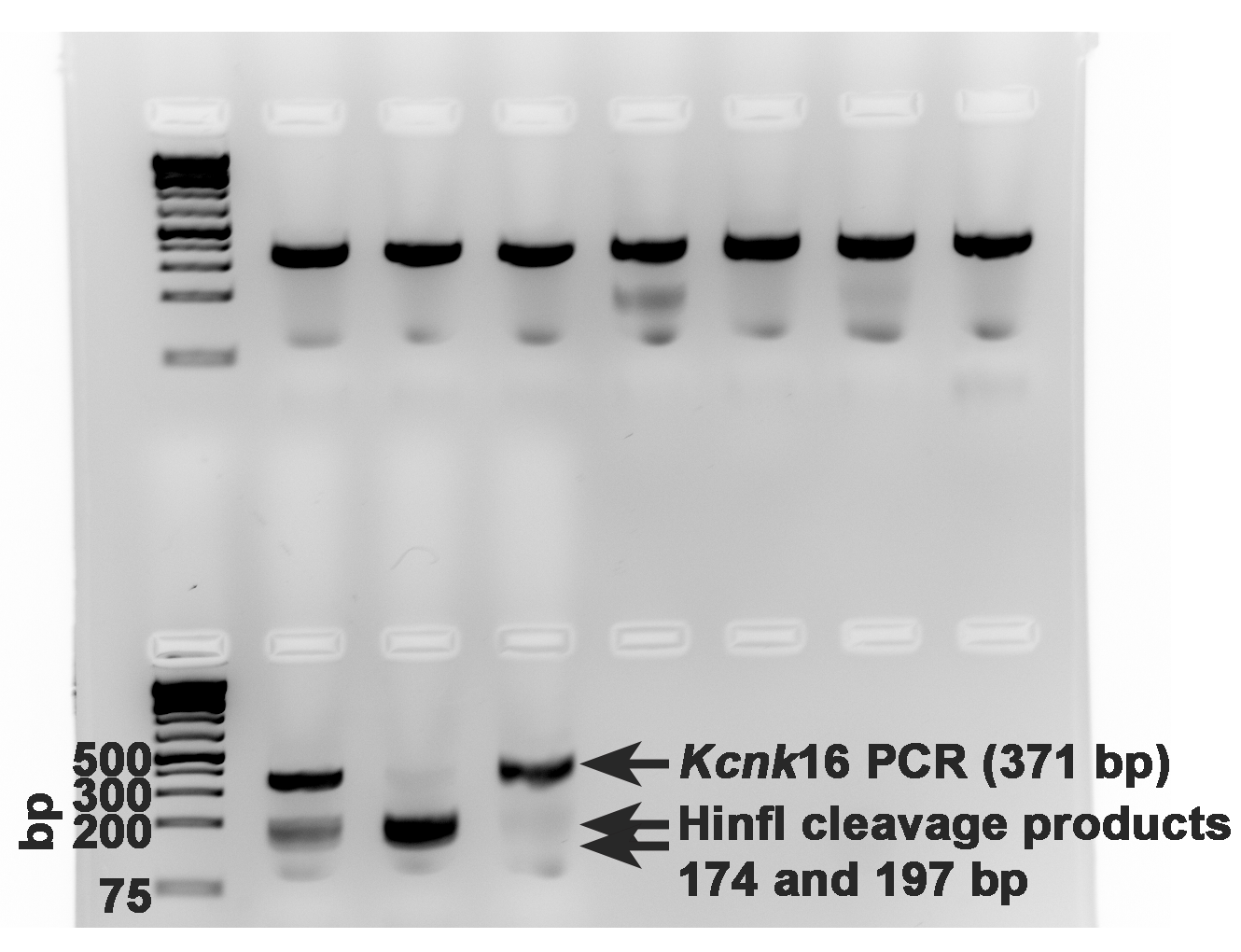

Supplement: Figure 1—figure supplement 1—source data 2. [file elife-89967-fig1-figsupp1-data2.tif]
