# Supplementary material for: The MODY-associated KCNK16 L114P mutation increases islet glucagon secretion and limits insulin secretion resulting in transient neonatal diabetes and glucose dyshomeostasis in adults
Source: eLife. 2024 May 3;12:RP89967. doi: 10.7554/eLife.89967 (PMC11068355; doi:10.7554/eLife.89967)
Supplement: Supplementary file 1. [file elife-89967-supp1.docx]

Supplementary File 1

| **Gene** | **Forward primer** | **Reverse primer** |
| --- | --- | --- |
| *18sRNA* | GTAACCCGTTGAACCCCATT | CCATCCAATCGGTAGTAGCG |
| *Cacna1g* | GAGACACAGAGTACGGGAGC | CAGGCATTTCATGGTCAGCG |
| *Sst* | CCACCGGGAAACAGGAACTG | TTGCTGGGTTCGAGTTGGC |
| *Asb11* | TGGTGGACTGTCAGACTGCT | ATTGACGTTGATGCCTTGCG |
| *Fxyd3* | ACTCTGCTTTCTCCCGGAAC | CTCGGAGGCTGTACCAATCATA |
| *Aldh1a3* | GGGTCACACTGGAGCTAGGA | CTGGCCTCTTCTTGGCGAA |
| *Camk1d* | CCGCCCTACAGCATTAGTCT | GAAAAGGCCCCAGTTCCGA |
| *Cxcl1* | ACCCAAACCGAAGTCATAGCC | TTGTCAGAAGCCAGCGTTCA |
| *Adcyap1r1* | CTGCGTGCAGAAATGCTACTG | AGCCGTAGAGTAATGGTGGATAG |
| *Aldob* | AGAAGGACAGCCAGGGAAAT | GTTCAGAGAGGCCATCAAGC |
| *Pdk4* | TGGTAGCAGTAGTCCAAGATGC | GTGGATTGGTTGGCCTGGAA |
| *Tgfb2* | TCGACATGGATCAGTTTATGCG | CCCTGGTACTGTTGTAGATGGA |
